# {5,15-Bis[3,5-bis­(6′-methyl-2,2′-bipyridyl-6-yl)phen­yl]-10,20-di­phenyl­porphyrin}zinc(II) 1,2-di­meth­oxy­ethane 0.296-solvate

**DOI:** 10.1107/S2414314626002592

**Published:** 2026-03-17

**Authors:** Aguri Akitomi, Yuta Kato, Yosuke Hosoya, Kosuke Sugawa, Joe Otsuki

**Affiliations:** a Department of Materials and Applied Chemistry College of Science and Technology Nihon University, 1-8-14 Kanda Surugadai Chiyoda Tokyo 101-8308 Japan; Purdue University, USA

**Keywords:** crystal structure, bi­pyridine, porphyrin

## Abstract

In the title compound, [Zn(C_88_H_60_N_12_)]·0.296C_4_H_10_O_2_, the porphyrin macrocycle is essentially planar, with the Zn atom located in the plane on an inversion centre. The non-substituted and bi­pyridine-bearing phenyl rings are twisted with respect to the porphyrin plane by 65.55 (6) and 64.85 (7)°, respectively. The phenyl and pyridine rings are moderately twisted, with dihedral angles of 21.21 (17) and 30.33 (14)°. The bi­pyridine moieties adopt an *s*-*trans* conformation with dihedral angles of 17.52 (18)° and 13.92 (13)°. The bi­pyridine units are located on both sides of the porphyrin plane due to the substitution at the 3- and 5-positions of the *meso-*phenyl ring. One bi­pyridine moiety folds back towards the porphyrin, while the other extends away from the porphyrin. π–π inter­actions between the porphyrin plane and the terminal pyridine ring of the extended bi­pyridine propagate along the [011] and [011] directions within the *bc* plane. The folded bi­pyridine units project from the network, resulting in alternating layers. The folded bi­pyridine layer contains void channels that are partially occupied by the crystallization solvent, 1,2-di­meth­oxy­ethane.

## Abstract 2

In the title compound, the porphyrin macrocycle is essentially planar, with the Zn atom located in the plane on an inversion centre. The bi­pyridine units are located on both sides of the porphyrin plane due to the substitution at the 3- and 5-positions of the *meso-*phenyl ring. One bi­pyridine moiety folds back towards the porphyrin, while the other extends away from the porphyrin.

## Graphical abstract

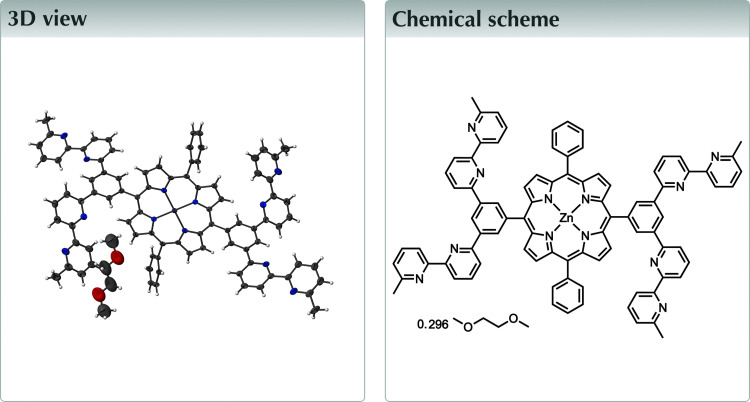

## Structure description

Porphyrins are characterized by their excellent visible-light absorption and fluorescence properties arising from extensive π-conjugation over the mol­ecular framework, as well as by their ability to accommodate metal ions at the central core (Otsuki *et al.*, 2018 ▸; Nikolaou *et al.*, 2024 ▸; Ganjali *et al.*, 2025 ▸). Porphyrins are promising building blocks for the construction of supra­molecular assemblies for functional materials. One strategy to assemble porphyrins is to use metal coordination inter­actions. For example, we have developed metal-driven assembly of porphyrins bearing bi­pyridine (Otsuki *et al.*, 2024 ▸) or phenanthroline (Otsuki *et al.*, 2025 ▸) ligands at the 3-positions of the *meso*-phenyl rings. We have newly prepared zinc(II) 5,15-bis­[3,5-bis­(6′-methyl-2,2′-bipyridin-6-yl)phen­yl]-10,20-di­phenyl­porphyrin [Por(Phbpy_2_)_2_] with bi­pyridine moieties at the 3- and 5-positions of two of the *meso*-phenyl rings).

**Figure 1:**
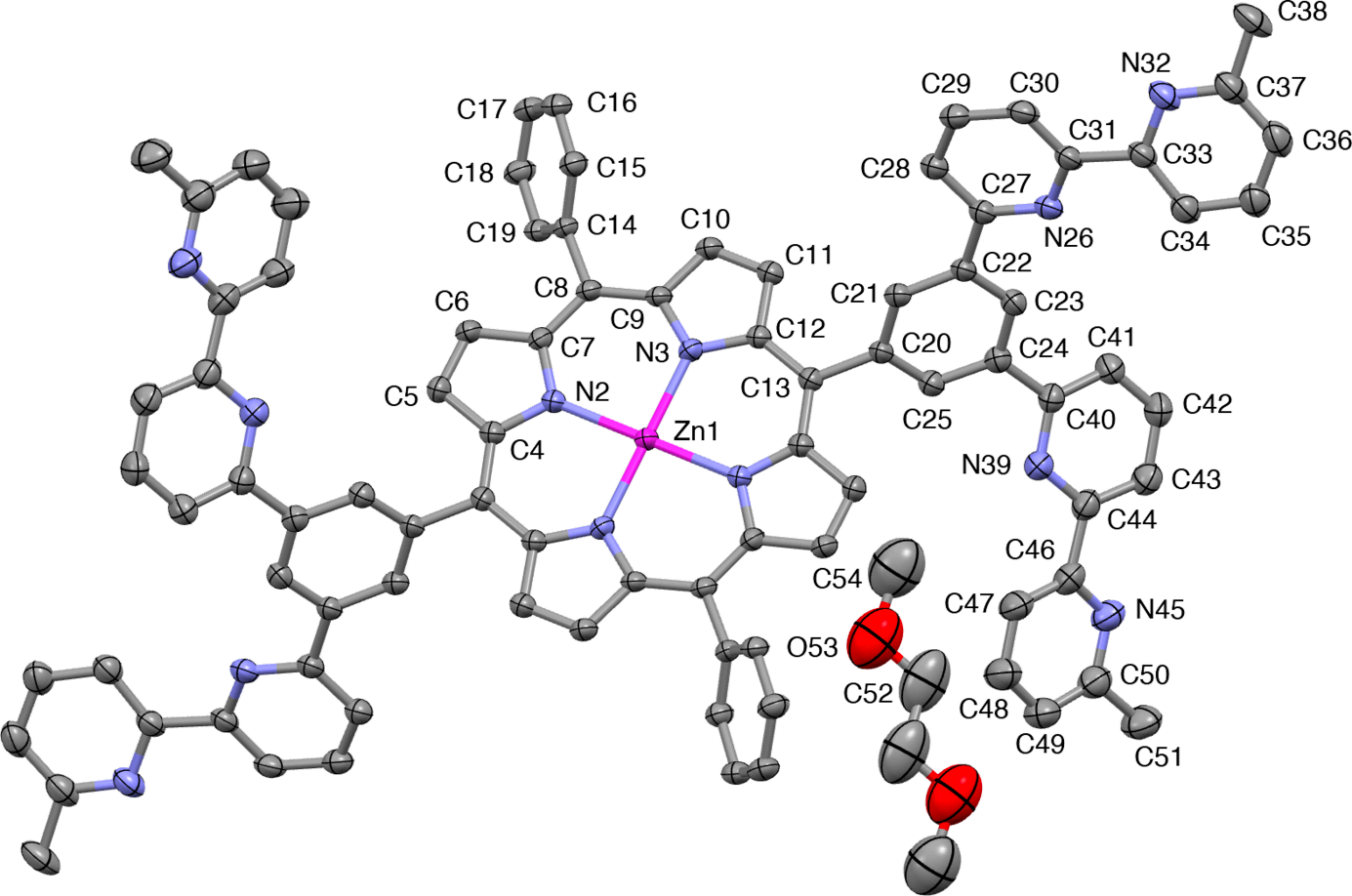
Crystal structure (displacement ellipsoid representation with displacement ellipsoids drawn at the 50% probability level) of [Por(Phbpy_2_)_2_]. Hydrogen atoms are omitted for clarity. Unlabelled atoms are created by inversion (symmetry operator: −*x*, 1 − *y*, 1 − *z*).

The molecular entities of [Por(Phbpy_2_)_2_] are shown in Fig. 1 ▸. The porphyrin macrocycle, as defined by 20 C and 4 N atoms, is essentially planar with a root mean square deviation from the least-squares plane of 0.0384 Å. The Zn atom is located in the plane on an inversion centre. The dihedral angle between the least-squares porphyrin plane and the non-substituted phenyl (C14–C19) plane is 65.55 (6)°, while that with the bi­pyridine-bearing phenyl (C20–C25) plane is 64.85 (7)°. The bi­pyridine units are located on opposite sides of the porphyrin plane due to substitution at the 3- and 5-positions of the *meso*-phenyl rings. Both 2,2′-bi­pyridine moieties adopt an expected *s*-*trans* conformation. One bi­pyridine unit extends away from the porphyrin centre, while the other bi­pyridine unit is folded back towards the porphyrin centre. For the extended bi­pyridine unit, the phenyl ring and the bonded pyridine ring are twisted by 21.21 (17)° and the two pyridyl planes are twisted moderately with a dihedral angle of 17.52 (18)°. The corresponding angles for the folded bi­pyridine unit are 30.33 (14) and 13.92 (13)°.

**Figure 2:**
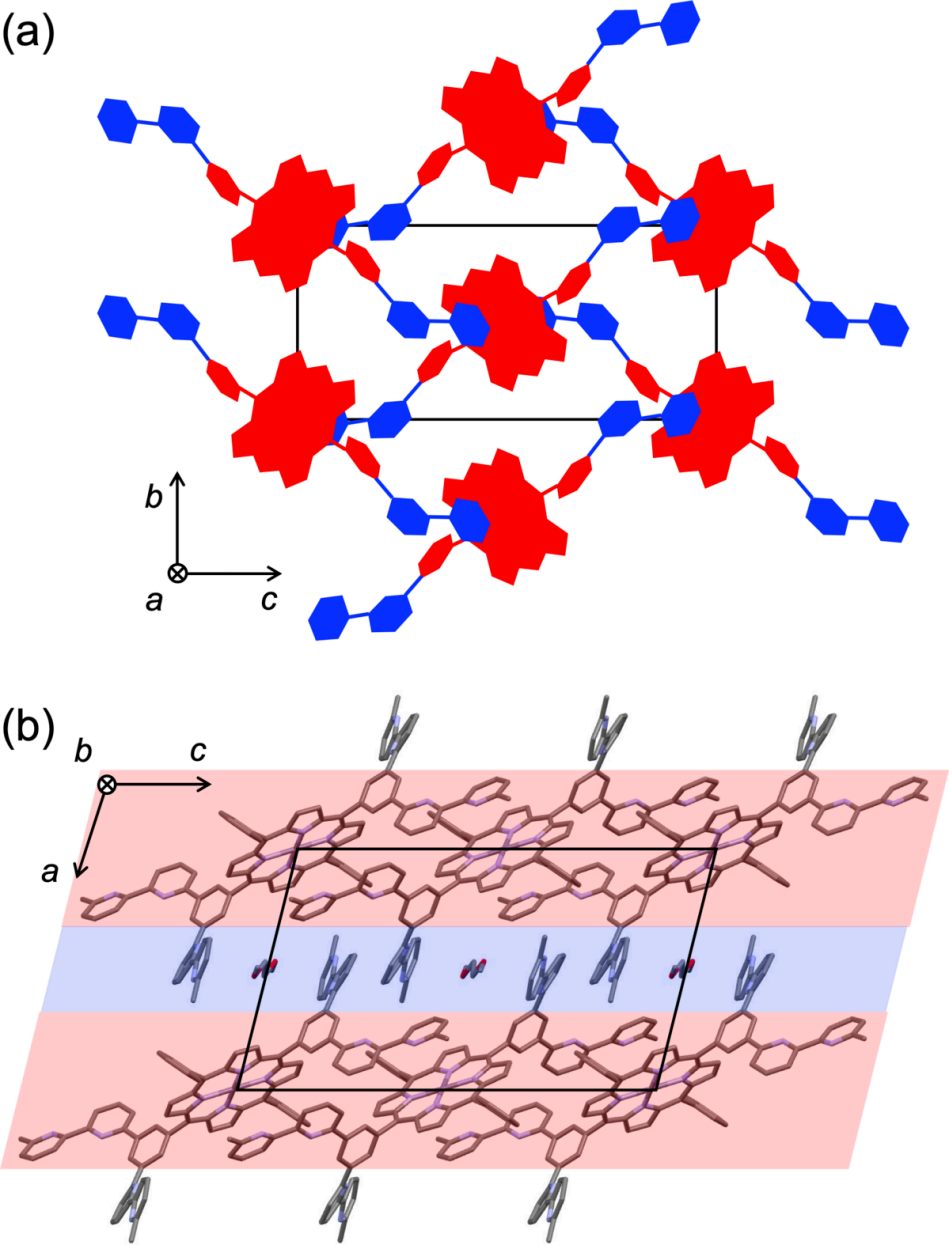
Crystal packing. (*a*) Two-dimensional network formed from π–π stacking between the porphyrin plane (red) and extended bi­pyridine units (blue). The folded bi­pyridine units and unsubstituted phenyl moieties are omitted for clarity. (*b*) Alternate layers of the two-dimensional network (light red) and folded bi­pyridine units (light blue).

The crystal packing (Fig. 2 ▸) features π–π inter­actions between the porphyrin macrocycle and the terminal pyridine ring of the extended bi­pyridine unit of neighboring mol­ecules. The two planes are nearly parallel, with a dihedral angle of 5.02 (17)°. The distances from the porphyrin least-squares plane to the pyridine C and N atoms range from 3.18 to 3.40 Å, consistent with π–π inter­actions. The shortest inter­molecular contact is 3.2635 (42) Å between the pyrrole α-carbon atom (C5) and atom C40 at the 3-position of the terminal pyridine ring (symmetry operation: *x*, 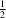 − *y*, 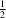 + *z*). These inter­actions occur on both sides of the porphyrin plane and propagate along both the [011] and [01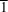] directions, generating a mesh network. The folded bi­pyridine units project from the two-dimensional network, resulting in alternating layers of the π-π stacked framework and protruding bi­pyridine moieties. Void channels run parallel to (010) between the folded bi­pyridine units and are partially occupied by disordered 1,2-di­meth­oxy­ethane solvent mol­ecules.

## Synthesis and crystallization

Compound **1** (Ono *et al.*, 2014 ▸) and 6-bromo-6′-methyl-2,2′-bi­pyridine (Bianchini *et al.*, 2007 ▸; Patroniak *et al.*, 2005 ▸) were prepared according literature procedures.

Compound **2**. A solution of 1 (205 mg, 0.570 mmol) in pyrrole (1.60 ml, 22.8 mmol) was degassed by bubbling N_2_ for 10 min, then tri­fluoro­acetic acid (5 µl, 6 µmol) was added. The solution was stirred for 40 min at room temperature, at which time no TLC spot corresponding to the starting aldehyde was detected by 2,4-di­nitro­phenyl­hydrazine. After tri­ethyl­amine (TEA) (12 µl, 86 µmol) was added to quench the reaction, the mixture was diluted with ethyl acetate (5 ml), washed with saturated NaCl aqueous solution, and dried over Na_2_SO_4_. The solvent was removed under reduced pressure to give a red oil. This material was purified by column chromatography (SiO_2_, CHCl_3_/MeOH/TEA = 97/3/1) followed by GPC to afford a yellow solid (139 mg, 0.293 mmol, 51%). HRMS (LDI) *m*/*z*: [*M* – H]^+^ (C_27_H_35_B_2_O_4_N_2_^+^) calculated 473.2778; found 473.2777. ^1^H NMR [CDCl_3_, *δ* (p.p.m.)]: 8.18 (*s*, 1H), 7.96 (*bs*, 2H), 7.80 (*s*, 2H), 6.68 (*m*, 2H), 6.14 (*m*, 2H), 5.92 (*m*, 2H), 5.47 (*s*, 1H), 1.32 (*s*, 24H). ^13^C NMR [CDCl_3_, *δ* (p.p.m.)]: 140.52, 140.05, 137.53, 132.47, 117.27, 108.10, 107.134, 77.20, 83.71, 44.20, 24.77.

**3-H_2_**. Benzaldehyde (2.04 g, 19.3 mmol) was added to a solution of **2** (9.15 g, 19.3 mmol) and NH_4_Cl (10.3 g, 193 mmol) in aceto­nitrile (1.92 l) cooled in an ice bath. The mixture was degassed by bubbling nitro­gen for 40 min, then BF_3_OEt_2_ (218 µl, 1.77 mmol) was added. The mixture was stirred at 2 °C for 4.5 h. Then 2,3-di­chloro-5,6-di­cyano-*p*-quinodi­methane (6.54 g, 29.1 mmol) was added to the solution, which was further stirred at room temperature for 1 h. TEA (9.96 ml, 71.7 mmol) was added to quench the reaction. The mixture was diluted with CHCl_3_, washed with a saturated aqueous NaCl solution, and dried over Na_2_SO_4_. The solvent was removed under reduced pressure. The residue was dissolved in CHCl_3_ and passed through a SiO_2_ pad. The solvent was removed under reduced pressure to give a black solid. This material was washed by deca­ntation with hexane, methanol, acetone, and aceto­nitrile, yielding a purple solid (551 mg, 0.492 mmol, 5.1%). HRMS (LDI) *m*/*z*: [*M*]^+^ (C_68_H_74_B_4_N_4_O_8_^+^) calculated 1118.5879; found 1118.5768. ^1^H NMR [CDCl_3_, *δ* (p.p.m.)]: 8.84 (*d*, *J* = 4.6 Hz, 4H), 8.81 (*d*, *J* = 4.6 Hz, 4H), 8.72 (*s*, 4H), 8.68 (*m*, 2H), 8.21 (*dd*, *J* = 7.1 Hz, *J* = 2.4 Hz, 4H), 7.74 (*m*, 6H), 1.36 (*s*, 48H), −2.80 (*s*, 2H). ^13^C NMR [CDCl_3_, *δ* (p.p.m.)]: 143.01, 142.42 141.15, 140.61, 134.64, 131.12, 127.78, 126.76, 120.44, 120.02, 84.16, 25.06.

**3-Zn**. A solution of **3-H_2_** (519 mg, 0.464 mmol) in CHCl_3_ (47 ml) and a solution of Zn(OAc)_2_·2(H_2_O) (660 mg, 3.01 mmol) in MeOH (9.4 ml) were mixed and stirred at room temperature for 4 h. The solution was washed with a saturated aqueous NaCl solution and dried over Na_2_SO_4_. The solvent was removed under reduced pressure and dried under vacuum to give a pink solid (521 mg, 0.441 mg, 95%). HRMS (LDI) *m*/*z*: [*M* + H]^+^ (C_68_H_74_B_4_N_4_O_8_Zn^+^) calculated 1180.5014; found 1180.5132. ^1^H NMR [CDCl_3_, *δ* (p.p.m.)]: 8.85 (*d*, *J* = 4.6 Hz, 4H), 8.82 (*d*, *J* = 5.0 Hz, 4H), 8.66 (*d*, *J* = 0.9 Hz, 4H), 8.61 (*t*, *J* = 0.9 Hz, 2H), 8.14 (*dd*, *J* = 7.3 Hz, 1.8 Hz, 4H), 7.67 (*m*, 6H), 1.29 (*s*, 48H). ^13^C NMR [CDCl_3_, *δ* (p.p.m.)]: 149.15, 141.79, 140.61, 139.13, 133.33, 131.29, 130.74, 126.35, 125.55, 20.12, 119.93, 82.82, 23.87.

**Figure 3:**
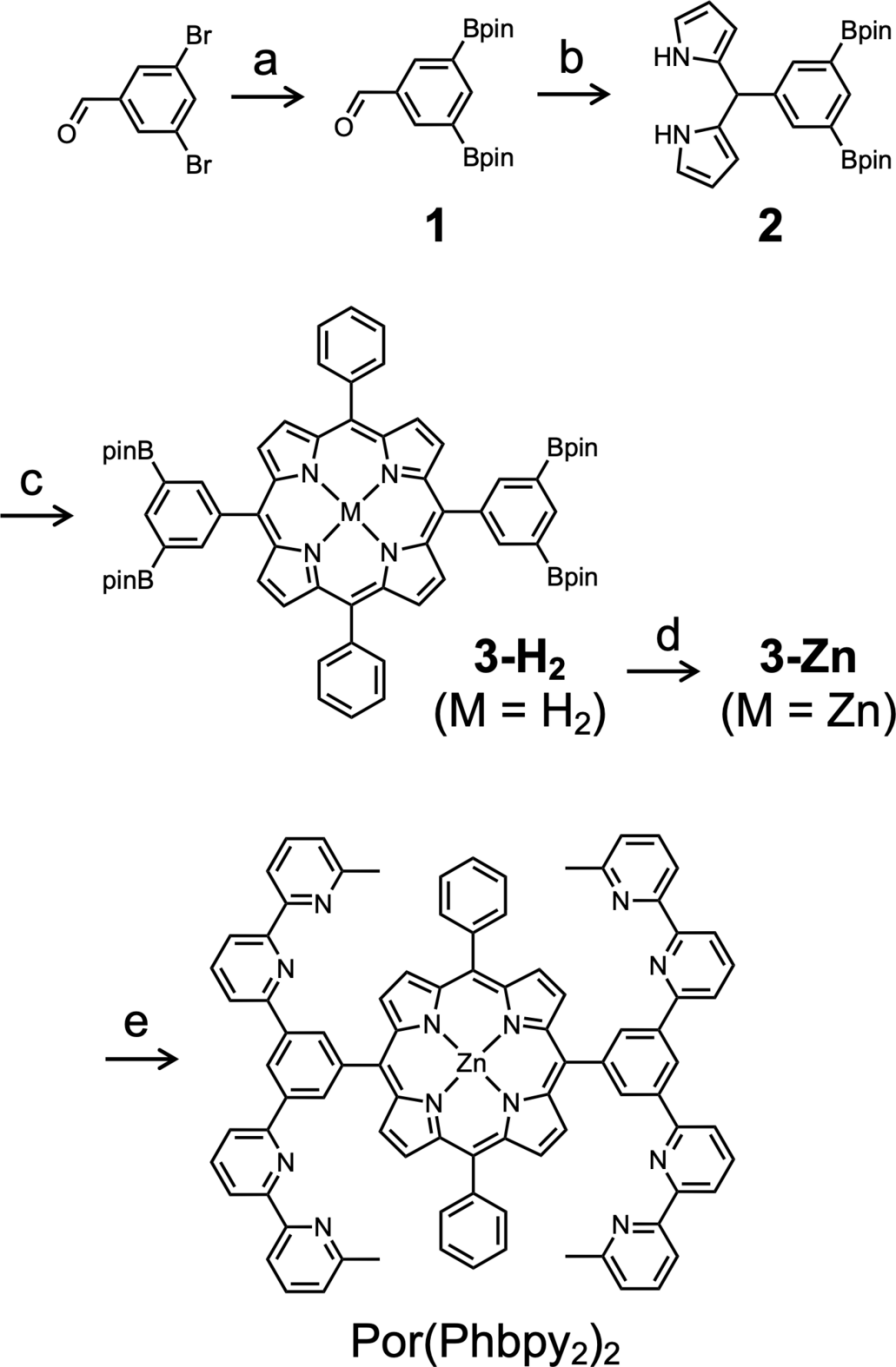
Preparation of [Por(Phbpy_2_)_2_]. (*a*) (Bpin)_2_, PdCl_2_(PPh_3_)_2_, dioxane, 80 °C, 21 h; (*b*) pyrrole, TFA, CH_2_Cl_2_, r.t., 1 h, 34% in 2 steps; (*c*) i) PhCHO, NH_4_Cl, BF_3_OEt_2_, MeCN, 0 °C, 4.5 h; ii) DDQ, r.t., 1 h, 6%; (*d*) Zn(OAc)_2_·2(H_2_O), CHCl_3_, MeOH, r.t., 4 h, 95%; (*e*) 6-bromo-6′-methyl-2,2′-bi­pyridine, Pd(PPh_3_)_4_, toluene, EtOH, Na_2_CO_3_, H_2_O, reflux, 26 h, 31%.

**Por(Phbpy_2_)_2_.** A solution of **3-Zn** (300 mg, 0.254 mmol), 6-bromo-6′-methyl-2,2′-bi­pyridine (317 mg, 1.27 mmol), and Pd(PPh_3_)_4_ (26 mg, 220 µmol) in toluene (50 ml) and EtOH (20 ml) and a solution of Na_2_CO_3_ (707 mg, 6.69 mmol) in H_2_O (12 ml) were mixed and degassed with three freeze–pump–thaw cycles, then refluxed for 26 h. The mixture was diluted with CHCl_3_, washed with a saturated aqueous NaCl solution, and dried over Na_2_SO_4_. The solvent was removed under reduced pressure to give a purple solid. The residue was dissolved in CHCl_3_ and passed through a SiO_2_ pad. The solvent was removed under reduced pressure to give a pink solid, which was washed by deca­ntation with hexane, methanol, acetone, and chloro­form, yielding a pink solid (108 mg, 0.0800 mmol, 31%). HRMS (LDI) *m*/*z*: [*M* + H]^+^ (C_88_H_61_N_12_Zn^+^) calculated 1349.4355; found 1349.4376. ^1^H NMR [CDCl_3_, *δ* (p.p.m.)]: 9.46 (*t*, *J* = 1.8 Hz, 2H), 9.18 (*d*, *J* = 4.6 Hz, 4H), 9.09 (*d*, *J* = 1.8 Hz, 4H), 9.03 (*d*, *J* = 4.6 Hz, 4H), 8.51–8.44 (*m*, 8H), 8.25 (*m*, 4H), 8.10 (*d*, *J* = 7.8 Hz, 4H), 7.96 (*t*, *J* = 7.8 Hz, 4H), 7.75 (*m*, 6H), 7.60 (*t*, *J* = 7.8 Hz, 4H), 7.13 (*d*, *J* = 7.3 Hz, 4H), 2.62 (*s*, 12H). See Fig. 3 ▸ for the synthesis.

Our intention was to crystallize Ag^+^ complexes of [Por(Phbpy_2_)_2_] and so we dissolved silver tri­fluoro­methane­sulfonate in the crystallization solution. A 100 µ*M* solution of [Por(Phbpy_2_)_2_] containing 1 equivalent AgOTf in DME/THF (7:3, *v*/*v*), after sonication and filtration through Chromatdisc, was allowed to evaporate slowly from a pinhole in the lid. After a month, rhombic crystals were obtained. No coordination of Ag^+^ to the bi­pyridine ligands was observed in the crystal structure.

## Refinement

Crystal data, data collection and structure refinement details are summarized in Table 1 ▸. The solvent mol­ecule (1,2-di­meth­oxy­ethane) was refined with its occupancy factor as a free variable, converging to 0.296 (11). Due to disorder and weak electron density, geometric restraints (DFIX and DANG) were applied to maintain reasonable C—O and C—C distances, and SIMU and RIGU restraints were applied to stabilize anisotropic displacement parameters. The maximum (0.99 e Å^−3^) and minimum (–0.80 e Å^−3^) residual electron densities are located in the vicinity of the Zn atom and are attributed to Fourier series truncation effects.

## Supplementary Material

Crystal structure: contains datablock(s) I. DOI: 10.1107/S2414314626002592/zl4093sup1.cif

Structure factors: contains datablock(s) I. DOI: 10.1107/S2414314626002592/zl4093Isup3.hkl

CCDC reference: 2536703

Additional supporting information:  crystallographic information; 3D view; checkCIF report

## Figures and Tables

**Table 1:** Experimental details

Crystal data	
Chemical formula	[Zn(C_88_H_60_N_12_)]·0.296C_4_H_10_O_2_
*M*_r_	1377.44
Crystal system, space group	Monoclinic, *P*2_1_/*c*
Temperature (K)	93
*a*, *b*, *c* (Å)	14.3759 (5), 10.9721 (3), 24.1950 (7)
β (°)	104.427 (3)
*V* (Å^3^)	3696.0 (2)
*Z*	2
Radiation type	Cu *K*α
μ (mm^−1^)	0.90
Crystal size (mm)	0.30 × 0.17 × 0.05

Data collection	
Diffractometer	XtaLAB AFC10 (RCD3): quarter-chi single
Absorption correction	Multi-scan (*CrysAlis PRO*; Rigaku OD, 2025 ▸)
*T*_min_, *T*_max_	0.381, 1.000
No. of measured, independent and observed [*I* > 2σ(*I*)] reflections	26839, 7160, 5282
*R*_int_	0.085
(sin θ/λ)_max_ (Å^−1^)	0.622

Refinement	
*R*[*F*^2^ > 2σ(*F*^2^)], *wR*(*F*^2^), *S*	0.069, 0.202, 1.03
No. of reflections	7160
No. of parameters	488
No. of restraints	23
H-atom treatment	H-atom parameters constrained
Δρ_max_, Δρ_min_ (e Å^−3^)	0.99, −0.81

Computer programs: *CrysAlis PRO* (Rigaku OD, 2025 ▸), *SHELXT* (Sheldrick, 2015*a* ▸), *SHELXL2018/3* (Sheldrick, 2015*b* ▸), *OLEX2* (Dolomanov *et al.*, 2009 ▸) and *Mercury* (Macrae *et al.*, 2020 ▸).

## full crystallographic data

### {5,15-Bis[3,5-bis(6'-methyl-2,2'-bipyridyl-6-yl)phenyl]-10,20-diphenylporphyrin}zinc(II) 1,2-dimethoxyethane 0.296-solvate Crystal data

**Table:**

[Zn(C_88_H_60_N_12_)]·0.296C_4_H_10_O_2_	*F*(000) = 1434
*M**_r_* = 1377.44	*D*_x_ = 1.238 Mg m^−^^3^
Monoclinic, *P*2_1_/*c*	Cu *K*α radiation, λ = 1.54184 Å
*a* = 14.3759 (5) Å	Cell parameters from 10737 reflections
*b* = 10.9721 (3) Å	θ = 3.8–72.4°
*c* = 24.1950 (7) Å	µ = 0.90 mm^−^^1^
β = 104.427 (3)°	*T* = 93 K
*V* = 3696.0 (2) Å^3^	Plate, clear dark red
*Z* = 2	0.3 × 0.17 × 0.05 mm

### {5,15-Bis[3,5-bis(6'-methyl-2,2'-bipyridyl-6-yl)phenyl]-10,20-diphenylporphyrin}zinc(II) 1,2-dimethoxyethane 0.296-solvate Data collection

**Table:**

XtaLAB AFC10 (RCD3): quarter-chi single diffractometer	7160 independent reflections
Radiation source: Rotating-anode X-ray tube (dual wavelength), Rigaku (Cu) X-ray DW Source	5282 reflections with *I* > 2σ(*I*)
Mirror monochromator	*R*_int_ = 0.085
Detector resolution: 5.8140 pixels mm^-1^	θ_max_ = 73.6°, θ_min_ = 3.2°
ω scans	*h* = −17→17
Absorption correction: multi-scan (CrysAlisPro; Rigaku OD, 2025)	*k* = −12→12
*T*_min_ = 0.381, *T*_max_ = 1.000	*l* = −29→29
26839 measured reflections	

### {5,15-Bis[3,5-bis(6'-methyl-2,2'-bipyridyl-6-yl)phenyl]-10,20-diphenylporphyrin}zinc(II) 1,2-dimethoxyethane 0.296-solvate Refinement

**Table:**

Refinement on *F*^2^	Primary atom site location: dual
Least-squares matrix: full	Hydrogen site location: inferred from neighbouring sites
*R*[*F*^2^ > 2σ(*F*^2^)] = 0.069	H-atom parameters constrained
*wR*(*F*^2^) = 0.202	*w* = 1/[σ^2^(*F*_o_^2^) + (0.1281*P*)^2^ + 1.5094*P*] where *P* = (*F*_o_^2^ + 2*F*_c_^2^)/3
*S* = 1.03	(Δ/σ)_max_ = 0.001
7160 reflections	Δρ_max_ = 0.99 e Å^−^^3^
488 parameters	Δρ_min_ = −0.80 e Å^−^^3^
23 restraints	

### {5,15-Bis[3,5-bis(6'-methyl-2,2'-bipyridyl-6-yl)phenyl]-10,20-diphenylporphyrin}zinc(II) 1,2-dimethoxyethane 0.296-solvate Special details

**Table:**

Geometry. All esds (except the esd in the dihedral angle between two l.s. planes) are estimated using the full covariance matrix. The cell esds are taken into account individually in the estimation of esds in distances, angles and torsion angles; correlations between esds in cell parameters are only used when they are defined by crystal symmetry. An approximate (isotropic) treatment of cell esds is used for estimating esds involving l.s. planes.

### {5,15-Bis[3,5-bis(6'-methyl-2,2'-bipyridyl-6-yl)phenyl]-10,20-diphenylporphyrin}zinc(II) 1,2-dimethoxyethane 0.296-solvate Fractional atomic coordinates and isotropic or equivalent isotropic displacement parameters (Å^2^)

**Table:**

	*x*	*y*	*z*	*U*_iso_*/*U*_eq_	Occ. (<1)
Zn1	0.000000	0.500000	0.500000	0.03148 (19)	
N2	−0.04034 (17)	0.4322 (2)	0.56919 (10)	0.0298 (5)	
N3	0.06978 (17)	0.3406 (2)	0.49326 (10)	0.0309 (5)	
C4	−0.0969 (2)	0.4891 (3)	0.59936 (12)	0.0311 (6)	
C5	−0.1088 (2)	0.4109 (3)	0.64476 (12)	0.0347 (6)	
H5	−0.144400	0.428738	0.671982	0.042*	
C6	−0.0597 (2)	0.3073 (3)	0.64147 (12)	0.0338 (6)	
H6	−0.054961	0.238370	0.665800	0.041*	
C7	−0.0159 (2)	0.3202 (3)	0.59448 (11)	0.0306 (6)	
C8	0.0424 (2)	0.2333 (3)	0.57763 (12)	0.0308 (6)	
C9	0.0828 (2)	0.2440 (2)	0.53099 (12)	0.0300 (6)	
C10	0.1487 (2)	0.1579 (3)	0.51639 (12)	0.0339 (6)	
H10	0.170828	0.084211	0.535912	0.041*	
C11	0.1728 (2)	0.2016 (3)	0.46982 (12)	0.0338 (6)	
H11	0.214418	0.163708	0.450032	0.041*	
C12	0.1240 (2)	0.3165 (3)	0.45547 (12)	0.0304 (6)	
C13	0.1347 (2)	0.3927 (3)	0.41092 (12)	0.0306 (6)	
C14	0.0662 (2)	0.1199 (3)	0.61304 (12)	0.0308 (6)	
C15	0.0369 (2)	0.0059 (3)	0.58991 (13)	0.0355 (7)	
H15	−0.003010	0.000357	0.552267	0.043*	
C16	0.0653 (2)	−0.0994 (3)	0.62107 (13)	0.0385 (7)	
H16	0.046027	−0.176738	0.604472	0.046*	
C17	0.1218 (2)	−0.0920 (3)	0.67650 (14)	0.0396 (7)	
H17	0.141393	−0.164232	0.697865	0.048*	
C18	0.1495 (2)	0.0206 (3)	0.70064 (14)	0.0394 (7)	
H18	0.186873	0.026079	0.738922	0.047*	
C19	0.1225 (2)	0.1259 (3)	0.66874 (12)	0.0342 (6)	
H19	0.142751	0.202995	0.685230	0.041*	
C20	0.1907 (2)	0.3434 (3)	0.37156 (12)	0.0309 (6)	
C21	0.1576 (2)	0.2413 (3)	0.33828 (12)	0.0321 (6)	
H21	0.100354	0.202191	0.341749	0.038*	
C22	0.2069 (2)	0.1956 (3)	0.29995 (12)	0.0315 (6)	
C23	0.2909 (2)	0.2543 (3)	0.29519 (13)	0.0340 (6)	
H23	0.325477	0.223623	0.269448	0.041*	
C24	0.3244 (2)	0.3573 (3)	0.32783 (12)	0.0344 (6)	
C25	0.2742 (2)	0.3999 (3)	0.36613 (12)	0.0343 (6)	
H25	0.297528	0.469099	0.389011	0.041*	
N26	0.19960 (18)	0.0829 (2)	0.21372 (10)	0.0349 (6)	
C27	0.1683 (2)	0.0916 (3)	0.26160 (12)	0.0338 (6)	
C28	0.1016 (2)	0.0097 (3)	0.27355 (13)	0.0360 (7)	
H28	0.081807	0.015647	0.308104	0.043*	
C29	0.0648 (2)	−0.0805 (3)	0.23412 (13)	0.0391 (7)	
H29	0.018728	−0.136591	0.241114	0.047*	
C30	0.0956 (2)	−0.0882 (3)	0.18453 (13)	0.0391 (7)	
H30	0.070310	−0.148565	0.156707	0.047*	
C31	0.1645 (2)	−0.0059 (3)	0.17607 (13)	0.0354 (7)	
N32	0.1879 (2)	−0.1179 (3)	0.09536 (11)	0.0432 (6)	
C33	0.2022 (2)	−0.0117 (3)	0.12410 (13)	0.0377 (7)	
C34	0.2520 (2)	0.0853 (3)	0.10818 (13)	0.0414 (7)	
H34	0.259173	0.160094	0.128599	0.050*	
C35	0.2912 (3)	0.0699 (4)	0.06147 (14)	0.0474 (8)	
H35	0.325969	0.134078	0.049439	0.057*	
C36	0.2787 (3)	−0.0402 (4)	0.03294 (15)	0.0504 (9)	
H36	0.306282	−0.053257	0.001585	0.060*	
C37	0.2256 (3)	−0.1318 (3)	0.05037 (14)	0.0474 (8)	
C38	0.2096 (3)	−0.2537 (4)	0.02098 (17)	0.0589 (10)	
H38A	0.247962	−0.315823	0.045656	0.088*	
H38B	0.229083	−0.249436	−0.015023	0.088*	
H38C	0.141438	−0.275295	0.013159	0.088*	
N39	0.40329 (19)	0.5479 (3)	0.32907 (11)	0.0394 (6)	
C40	0.4074 (2)	0.4268 (3)	0.31901 (13)	0.0363 (7)	
C41	0.4834 (2)	0.3742 (3)	0.30164 (14)	0.0425 (7)	
H41	0.485603	0.288572	0.296291	0.051*	
C42	0.5554 (2)	0.4483 (4)	0.29226 (15)	0.0463 (8)	
H42	0.607550	0.414411	0.279807	0.056*	
C43	0.5511 (2)	0.5720 (3)	0.30115 (14)	0.0453 (8)	
H43	0.599554	0.624583	0.294382	0.054*	
C44	0.4745 (2)	0.6188 (3)	0.32023 (14)	0.0412 (7)	
N45	0.5462 (2)	0.8181 (3)	0.33620 (12)	0.0474 (7)	
C46	0.4663 (2)	0.7512 (3)	0.33246 (15)	0.0455 (8)	
C47	0.3811 (3)	0.8000 (3)	0.33960 (17)	0.0512 (9)	
H47	0.326441	0.749753	0.337045	0.061*	
C48	0.3774 (3)	0.9229 (4)	0.35048 (19)	0.0587 (10)	
H48	0.319662	0.959002	0.354961	0.070*	
C49	0.4592 (3)	0.9932 (4)	0.35480 (18)	0.0562 (10)	
H49	0.458684	1.077868	0.362875	0.067*	
C50	0.5422 (3)	0.9373 (4)	0.34709 (16)	0.0519 (9)	
C51	0.6319 (3)	1.0099 (4)	0.3483 (2)	0.0625 (11)	
H51A	0.688318	0.964440	0.369388	0.094*	
H51B	0.628742	1.088342	0.367098	0.094*	
H51C	0.636965	1.024021	0.309140	0.094*	
C52	0.5237 (17)	0.9367 (13)	0.5024 (9)	0.122 (8)	0.296 (11)
H52A	0.530762	0.909961	0.464564	0.147*	0.296 (11)
H52B	0.588279	0.939812	0.529016	0.147*	0.296 (11)
O53	0.4679 (13)	0.8594 (19)	0.5216 (7)	0.129 (7)	0.296 (11)
C54	0.5029 (19)	0.733 (2)	0.5188 (12)	0.133 (9)	0.296 (11)
H54A	0.458762	0.689069	0.487762	0.199*	0.296 (11)
H54B	0.505948	0.691508	0.555050	0.199*	0.296 (11)
H54C	0.567036	0.734882	0.511630	0.199*	0.296 (11)

### {5,15-Bis[3,5-bis(6'-methyl-2,2'-bipyridyl-6-yl)phenyl]-10,20-diphenylporphyrin}zinc(II) 1,2-dimethoxyethane 0.296-solvate Atomic displacement parameters (Å^2^)

**Table:**

	*U*^11^	*U*^22^	*U*^33^	*U*^12^	*U*^13^	*U*^23^
Zn1	0.0483 (3)	0.0200 (3)	0.0322 (3)	0.0030 (2)	0.0214 (2)	0.0024 (2)
N2	0.0425 (12)	0.0203 (13)	0.0314 (12)	0.0006 (9)	0.0183 (10)	−0.0002 (9)
N3	0.0466 (13)	0.0211 (13)	0.0290 (12)	0.0008 (10)	0.0170 (10)	0.0038 (9)
C4	0.0431 (15)	0.0217 (15)	0.0323 (14)	−0.0001 (11)	0.0165 (12)	−0.0005 (11)
C5	0.0505 (16)	0.0276 (16)	0.0320 (14)	−0.0005 (12)	0.0215 (12)	0.0012 (11)
C6	0.0502 (16)	0.0207 (16)	0.0350 (15)	−0.0006 (12)	0.0192 (12)	0.0027 (11)
C7	0.0458 (15)	0.0213 (15)	0.0283 (13)	−0.0038 (11)	0.0158 (11)	0.0011 (10)
C8	0.0463 (15)	0.0175 (15)	0.0315 (14)	−0.0022 (11)	0.0151 (11)	0.0017 (10)
C9	0.0455 (15)	0.0160 (14)	0.0311 (14)	0.0006 (11)	0.0143 (11)	0.0010 (10)
C10	0.0495 (16)	0.0202 (15)	0.0357 (15)	0.0014 (12)	0.0174 (12)	0.0026 (11)
C11	0.0473 (16)	0.0233 (16)	0.0351 (15)	0.0023 (12)	0.0183 (12)	−0.0011 (11)
C12	0.0439 (15)	0.0206 (15)	0.0301 (14)	−0.0004 (11)	0.0158 (11)	−0.0006 (11)
C13	0.0422 (14)	0.0228 (15)	0.0314 (14)	−0.0004 (11)	0.0177 (11)	−0.0011 (11)
C14	0.0466 (15)	0.0185 (15)	0.0323 (14)	−0.0007 (11)	0.0190 (12)	0.0026 (11)
C15	0.0519 (17)	0.0250 (16)	0.0321 (15)	−0.0013 (12)	0.0154 (12)	0.0010 (11)
C16	0.0557 (18)	0.0204 (16)	0.0434 (17)	−0.0025 (12)	0.0195 (14)	−0.0003 (12)
C17	0.0533 (18)	0.0228 (16)	0.0450 (17)	0.0010 (13)	0.0166 (14)	0.0064 (12)
C18	0.0568 (18)	0.0260 (17)	0.0360 (16)	−0.0019 (13)	0.0129 (13)	0.0053 (12)
C19	0.0523 (17)	0.0190 (15)	0.0344 (15)	−0.0026 (12)	0.0168 (12)	−0.0003 (11)
C20	0.0449 (15)	0.0213 (15)	0.0308 (14)	0.0044 (11)	0.0177 (11)	0.0016 (11)
C21	0.0432 (15)	0.0221 (15)	0.0340 (14)	0.0018 (11)	0.0154 (12)	0.0013 (11)
C22	0.0455 (15)	0.0215 (15)	0.0309 (14)	0.0039 (11)	0.0159 (11)	0.0005 (11)
C23	0.0435 (15)	0.0286 (17)	0.0342 (14)	0.0049 (12)	0.0176 (12)	−0.0003 (11)
C24	0.0435 (15)	0.0280 (17)	0.0346 (15)	0.0016 (12)	0.0154 (12)	0.0016 (11)
C25	0.0470 (16)	0.0264 (16)	0.0330 (14)	0.0004 (12)	0.0167 (12)	−0.0010 (11)
N26	0.0467 (14)	0.0240 (14)	0.0361 (13)	0.0047 (10)	0.0145 (10)	−0.0009 (10)
C27	0.0469 (16)	0.0234 (16)	0.0343 (15)	0.0047 (12)	0.0162 (12)	−0.0002 (11)
C28	0.0495 (17)	0.0262 (17)	0.0353 (15)	0.0017 (12)	0.0164 (13)	−0.0006 (11)
C29	0.0529 (18)	0.0245 (17)	0.0416 (16)	−0.0016 (13)	0.0150 (13)	0.0002 (12)
C30	0.0557 (18)	0.0222 (16)	0.0396 (16)	0.0035 (13)	0.0123 (13)	−0.0035 (12)
C31	0.0503 (17)	0.0248 (16)	0.0336 (15)	0.0072 (12)	0.0153 (13)	−0.0021 (11)
N32	0.0563 (16)	0.0373 (16)	0.0371 (14)	0.0089 (12)	0.0134 (12)	−0.0079 (11)
C33	0.0511 (17)	0.0296 (18)	0.0342 (15)	0.0083 (13)	0.0141 (13)	−0.0024 (12)
C34	0.0532 (18)	0.0371 (19)	0.0355 (16)	0.0086 (14)	0.0141 (13)	0.0005 (13)
C35	0.057 (2)	0.047 (2)	0.0420 (18)	0.0120 (16)	0.0199 (15)	0.0056 (15)
C36	0.064 (2)	0.055 (2)	0.0379 (17)	0.0167 (17)	0.0222 (15)	0.0012 (16)
C37	0.060 (2)	0.045 (2)	0.0383 (17)	0.0166 (16)	0.0131 (14)	−0.0051 (14)
C38	0.080 (3)	0.049 (2)	0.047 (2)	0.0221 (19)	0.0140 (17)	−0.0153 (16)
N39	0.0473 (14)	0.0338 (15)	0.0410 (14)	−0.0018 (11)	0.0183 (11)	0.0006 (11)
C40	0.0429 (16)	0.0359 (19)	0.0338 (14)	−0.0027 (12)	0.0163 (12)	0.0003 (12)
C41	0.0479 (17)	0.040 (2)	0.0439 (17)	−0.0011 (14)	0.0198 (14)	−0.0051 (14)
C42	0.0460 (17)	0.049 (2)	0.0508 (19)	−0.0010 (15)	0.0248 (15)	−0.0014 (16)
C43	0.0449 (17)	0.046 (2)	0.0475 (18)	−0.0058 (14)	0.0174 (14)	0.0010 (15)
C44	0.0443 (16)	0.042 (2)	0.0402 (16)	−0.0042 (14)	0.0155 (13)	0.0040 (13)
N45	0.0529 (16)	0.0397 (18)	0.0504 (16)	−0.0080 (13)	0.0147 (13)	0.0041 (13)
C46	0.0505 (18)	0.042 (2)	0.0471 (19)	−0.0049 (14)	0.0181 (14)	0.0076 (14)
C47	0.054 (2)	0.036 (2)	0.067 (2)	−0.0046 (15)	0.0228 (17)	0.0044 (16)
C48	0.061 (2)	0.041 (2)	0.078 (3)	0.0015 (17)	0.026 (2)	0.0040 (18)
C49	0.073 (2)	0.037 (2)	0.062 (2)	−0.0041 (17)	0.0237 (19)	0.0006 (16)
C50	0.062 (2)	0.045 (2)	0.050 (2)	−0.0053 (17)	0.0171 (16)	0.0028 (16)
C51	0.067 (2)	0.047 (3)	0.074 (3)	−0.0131 (18)	0.018 (2)	−0.0007 (19)
C52	0.117 (15)	0.168 (17)	0.075 (11)	−0.016 (12)	0.011 (10)	0.035 (12)
O53	0.124 (12)	0.155 (14)	0.099 (10)	−0.019 (9)	0.011 (8)	0.024 (10)
C54	0.124 (18)	0.150 (16)	0.118 (17)	0.001 (14)	0.019 (14)	0.031 (16)

### {5,15-Bis[3,5-bis(6'-methyl-2,2'-bipyridyl-6-yl)phenyl]-10,20-diphenylporphyrin}zinc(II) 1,2-dimethoxyethane 0.296-solvate Geometric parameters (Å, º)

**Table:**

Zn1—N2	2.044 (2)	C28—C29	1.384 (4)
Zn1—N2^i^	2.044 (2)	C29—H29	0.9500
Zn1—N3^i^	2.043 (2)	C29—C30	1.381 (4)
Zn1—N3	2.043 (2)	C30—H30	0.9500
N2—C4	1.371 (3)	C30—C31	1.392 (5)
N2—C7	1.379 (4)	C31—C33	1.490 (4)
N3—C9	1.381 (3)	N32—C33	1.347 (4)
N3—C12	1.366 (3)	N32—C37	1.340 (4)
C4—C5	1.437 (4)	C33—C34	1.390 (5)
C4—C13^i^	1.403 (4)	C34—H34	0.9500
C5—H5	0.9500	C34—C35	1.393 (4)
C5—C6	1.351 (4)	C35—H35	0.9500
C6—H6	0.9500	C35—C36	1.381 (5)
C6—C7	1.437 (4)	C36—H36	0.9500
C7—C8	1.396 (4)	C36—C37	1.390 (6)
C8—C9	1.397 (4)	C37—C38	1.506 (5)
C8—C14	1.501 (4)	C38—H38A	0.9800
C9—C10	1.442 (4)	C38—H38B	0.9800
C10—H10	0.9500	C38—H38C	0.9800
C10—C11	1.347 (4)	N39—C40	1.355 (4)
C11—H11	0.9500	N39—C44	1.345 (4)
C11—C12	1.443 (4)	C40—C41	1.389 (4)
C12—C13	1.403 (4)	C41—H41	0.9500
C13—C20	1.493 (4)	C41—C42	1.380 (5)
C14—C15	1.391 (4)	C42—H42	0.9500
C14—C19	1.390 (4)	C42—C43	1.378 (5)
C15—H15	0.9500	C43—H43	0.9500
C15—C16	1.385 (4)	C43—C44	1.394 (5)
C16—H16	0.9500	C44—C46	1.493 (5)
C16—C17	1.386 (5)	N45—C46	1.347 (4)
C17—H17	0.9500	N45—C50	1.339 (5)
C17—C18	1.383 (4)	C46—C47	1.386 (5)
C18—H18	0.9500	C47—H47	0.9500
C18—C19	1.389 (4)	C47—C48	1.377 (6)
C19—H19	0.9500	C48—H48	0.9500
C20—C21	1.392 (4)	C48—C49	1.389 (6)
C20—C25	1.386 (4)	C49—H49	0.9500
C21—H21	0.9500	C49—C50	1.394 (6)
C21—C22	1.394 (4)	C50—C51	1.511 (5)
C22—C23	1.398 (4)	C51—H51A	0.9800
C22—C27	1.488 (4)	C51—H51B	0.9800
C23—H23	0.9500	C51—H51C	0.9800
C23—C24	1.395 (4)	C52—C52^ii^	1.538 (19)
C24—C25	1.389 (4)	C52—H52A	0.9900
C24—C40	1.476 (4)	C52—H52B	0.9900
C25—H25	0.9500	C52—O53	1.33 (3)
N26—C27	1.347 (4)	O53—C54	1.48 (3)
N26—C31	1.343 (4)	C54—H54A	0.9800
C27—C28	1.397 (4)	C54—H54B	0.9800
C28—H28	0.9500	C54—H54C	0.9800
			
C9···C34	3.264 (4)		
			
N2—Zn1—N2^i^	180.00 (15)	C29—C28—H28	120.6
N3—Zn1—N2	89.94 (9)	C28—C29—H29	120.3
N3^i^—Zn1—N2	90.06 (9)	C30—C29—C28	119.5 (3)
N3^i^—Zn1—N2^i^	89.94 (9)	C30—C29—H29	120.3
N3—Zn1—N2^i^	90.06 (9)	C29—C30—H30	120.6
N3—Zn1—N3^i^	180.00 (14)	C29—C30—C31	118.8 (3)
C4—N2—Zn1	126.68 (19)	C31—C30—H30	120.6
C4—N2—C7	106.8 (2)	N26—C31—C30	122.1 (3)
C7—N2—Zn1	126.51 (18)	N26—C31—C33	116.7 (3)
C9—N3—Zn1	126.38 (18)	C30—C31—C33	121.2 (3)
C12—N3—Zn1	126.29 (18)	C37—N32—C33	118.5 (3)
C12—N3—C9	106.7 (2)	N32—C33—C31	115.6 (3)
N2—C4—C5	109.5 (2)	N32—C33—C34	122.9 (3)
N2—C4—C13^i^	125.5 (3)	C34—C33—C31	121.5 (3)
C13^i^—C4—C5	125.0 (3)	C33—C34—H34	120.9
C4—C5—H5	126.4	C33—C34—C35	118.2 (3)
C6—C5—C4	107.1 (2)	C35—C34—H34	120.9
C6—C5—H5	126.4	C34—C35—H35	120.5
C5—C6—H6	126.2	C36—C35—C34	118.9 (3)
C5—C6—C7	107.6 (3)	C36—C35—H35	120.5
C7—C6—H6	126.2	C35—C36—H36	120.2
N2—C7—C6	109.0 (2)	C35—C36—C37	119.6 (3)
N2—C7—C8	125.8 (2)	C37—C36—H36	120.2
C8—C7—C6	125.2 (3)	N32—C37—C36	121.9 (3)
C7—C8—C9	125.4 (3)	N32—C37—C38	116.1 (3)
C7—C8—C14	117.8 (2)	C36—C37—C38	122.0 (3)
C9—C8—C14	116.7 (2)	C37—C38—H38A	109.5
N3—C9—C8	125.7 (3)	C37—C38—H38B	109.5
N3—C9—C10	109.3 (2)	C37—C38—H38C	109.5
C8—C9—C10	125.0 (3)	H38A—C38—H38B	109.5
C9—C10—H10	126.5	H38A—C38—H38C	109.5
C11—C10—C9	107.0 (3)	H38B—C38—H38C	109.5
C11—C10—H10	126.5	C44—N39—C40	118.0 (3)
C10—C11—H11	126.2	N39—C40—C24	114.0 (3)
C10—C11—C12	107.5 (3)	N39—C40—C41	122.3 (3)
C12—C11—H11	126.2	C41—C40—C24	123.7 (3)
N3—C12—C11	109.4 (2)	C40—C41—H41	120.5
N3—C12—C13	126.1 (3)	C42—C41—C40	119.0 (3)
C13—C12—C11	124.4 (3)	C42—C41—H41	120.5
C4^i^—C13—C20	117.9 (2)	C41—C42—H42	120.4
C12—C13—C4^i^	125.1 (3)	C43—C42—C41	119.3 (3)
C12—C13—C20	117.0 (3)	C43—C42—H42	120.4
C15—C14—C8	120.8 (3)	C42—C43—H43	120.5
C19—C14—C8	120.6 (3)	C42—C43—C44	119.1 (3)
C19—C14—C15	118.5 (3)	C44—C43—H43	120.5
C14—C15—H15	119.6	N39—C44—C43	122.3 (3)
C16—C15—C14	120.8 (3)	N39—C44—C46	115.5 (3)
C16—C15—H15	119.6	C43—C44—C46	122.2 (3)
C15—C16—H16	120.0	C50—N45—C46	117.9 (3)
C15—C16—C17	120.0 (3)	N45—C46—C44	115.8 (3)
C17—C16—H16	120.0	N45—C46—C47	123.1 (3)
C16—C17—H17	120.0	C47—C46—C44	121.1 (3)
C18—C17—C16	119.9 (3)	C46—C47—H47	120.6
C18—C17—H17	120.0	C48—C47—C46	118.7 (3)
C17—C18—H18	120.1	C48—C47—H47	120.6
C17—C18—C19	119.8 (3)	C47—C48—H48	120.5
C19—C18—H18	120.1	C47—C48—C49	119.0 (4)
C14—C19—H19	119.5	C49—C48—H48	120.5
C18—C19—C14	120.9 (3)	C48—C49—H49	120.6
C18—C19—H19	119.5	C48—C49—C50	118.8 (4)
C21—C20—C13	120.0 (3)	C50—C49—H49	120.6
C25—C20—C13	121.0 (3)	N45—C50—C49	122.5 (3)
C25—C20—C21	119.0 (3)	N45—C50—C51	116.1 (4)
C20—C21—H21	119.4	C49—C50—C51	121.4 (4)
C20—C21—C22	121.1 (3)	C50—C51—H51A	109.5
C22—C21—H21	119.4	C50—C51—H51B	109.5
C21—C22—C23	118.8 (3)	C50—C51—H51C	109.5
C21—C22—C27	121.2 (3)	H51A—C51—H51B	109.5
C23—C22—C27	119.8 (2)	H51A—C51—H51C	109.5
C22—C23—H23	119.7	H51B—C51—H51C	109.5
C24—C23—C22	120.7 (3)	C52^ii^—C52—H52A	110.1
C24—C23—H23	119.7	C52^ii^—C52—H52B	110.1
C23—C24—C40	120.9 (3)	H52A—C52—H52B	108.4
C25—C24—C23	119.2 (3)	O53—C52—C52^ii^	108 (3)
C25—C24—C40	119.7 (3)	O53—C52—H52A	110.1
C20—C25—C24	121.2 (3)	O53—C52—H52B	110.1
C20—C25—H25	119.4	C52—O53—C54	109.9 (18)
C24—C25—H25	119.4	O53—C54—H54A	109.5
C31—N26—C27	119.0 (3)	O53—C54—H54B	109.5
N26—C27—C22	116.0 (3)	O53—C54—H54C	109.5
N26—C27—C28	121.7 (3)	H54A—C54—H54B	109.5
C28—C27—C22	122.2 (3)	H54A—C54—H54C	109.5
C27—C28—H28	120.6	H54B—C54—H54C	109.5
C29—C28—C27	118.8 (3)		
			
Zn1—N2—C4—C5	179.77 (19)	C22—C23—C24—C25	1.2 (4)
Zn1—N2—C4—C13^i^	−2.5 (4)	C22—C23—C24—C40	−174.0 (3)
Zn1—N2—C7—C6	−179.38 (19)	C22—C27—C28—C29	176.3 (3)
Zn1—N2—C7—C8	1.0 (4)	C23—C22—C27—N26	−19.1 (4)
Zn1—N3—C9—C8	6.6 (4)	C23—C22—C27—C28	162.5 (3)
Zn1—N3—C9—C10	−170.61 (19)	C23—C24—C25—C20	−1.3 (4)
Zn1—N3—C12—C11	171.32 (19)	C23—C24—C40—N39	147.8 (3)
Zn1—N3—C12—C13	−6.2 (4)	C23—C24—C40—C41	−31.8 (5)
N2—C4—C5—C6	−0.3 (3)	C24—C40—C41—C42	177.2 (3)
N2—C7—C8—C9	−2.4 (5)	C25—C20—C21—C22	0.1 (4)
N2—C7—C8—C14	176.0 (3)	C25—C24—C40—N39	−27.3 (4)
N3—C9—C10—C11	−1.3 (3)	C25—C24—C40—C41	153.1 (3)
N3—C12—C13—C4^i^	5.1 (5)	N26—C27—C28—C29	−2.0 (5)
N3—C12—C13—C20	−174.8 (3)	N26—C31—C33—N32	162.2 (3)
C4—N2—C7—C6	0.6 (3)	N26—C31—C33—C34	−15.5 (4)
C4—N2—C7—C8	−179.1 (3)	C27—C22—C23—C24	175.3 (3)
C4—C5—C6—C7	0.6 (3)	C27—N26—C31—C30	0.9 (4)
C4^i^—C13—C20—C21	−116.8 (3)	C27—N26—C31—C33	−179.6 (3)
C4^i^—C13—C20—C25	61.1 (4)	C27—C28—C29—C30	0.8 (5)
C5—C6—C7—N2	−0.8 (3)	C28—C29—C30—C31	1.1 (5)
C5—C6—C7—C8	178.9 (3)	C29—C30—C31—N26	−2.0 (5)
C6—C7—C8—C9	178.0 (3)	C29—C30—C31—C33	178.5 (3)
C6—C7—C8—C14	−3.6 (4)	C30—C31—C33—N32	−18.3 (4)
C7—N2—C4—C5	−0.2 (3)	C30—C31—C33—C34	164.1 (3)
C7—N2—C4—C13^i^	177.6 (3)	C31—N26—C27—C22	−177.2 (3)
C7—C8—C9—N3	−1.7 (5)	C31—N26—C27—C28	1.1 (4)
C7—C8—C9—C10	175.2 (3)	C31—C33—C34—C35	175.4 (3)
C7—C8—C14—C15	117.3 (3)	N32—C33—C34—C35	−2.1 (5)
C7—C8—C14—C19	−66.3 (4)	C33—N32—C37—C36	0.1 (5)
C8—C9—C10—C11	−178.5 (3)	C33—N32—C37—C38	178.3 (3)
C8—C14—C15—C16	174.8 (3)	C33—C34—C35—C36	0.3 (5)
C8—C14—C19—C18	−176.2 (3)	C34—C35—C36—C37	1.6 (5)
C9—N3—C12—C11	−0.2 (3)	C35—C36—C37—N32	−1.8 (5)
C9—N3—C12—C13	−177.6 (3)	C35—C36—C37—C38	−179.9 (3)
C9—C8—C14—C15	−64.1 (4)	C37—N32—C33—C31	−175.7 (3)
C9—C8—C14—C19	112.3 (3)	C37—N32—C33—C34	1.9 (5)
C9—C10—C11—C12	1.1 (3)	N39—C40—C41—C42	−2.4 (5)
C10—C11—C12—N3	−0.6 (3)	N39—C44—C46—N45	165.7 (3)
C10—C11—C12—C13	176.9 (3)	N39—C44—C46—C47	−14.0 (5)
C11—C12—C13—C4^i^	−172.0 (3)	C40—C24—C25—C20	173.9 (3)
C11—C12—C13—C20	8.1 (4)	C40—N39—C44—C43	0.2 (5)
C12—N3—C9—C8	178.1 (3)	C40—N39—C44—C46	−179.6 (3)
C12—N3—C9—C10	0.9 (3)	C40—C41—C42—C43	0.9 (5)
C12—C13—C20—C21	63.1 (4)	C41—C42—C43—C44	0.9 (5)
C12—C13—C20—C25	−118.9 (3)	C42—C43—C44—N39	−1.6 (5)
C13^i^—C4—C5—C6	−178.1 (3)	C42—C43—C44—C46	178.2 (3)
C13—C20—C21—C22	178.1 (3)	C43—C44—C46—N45	−14.0 (5)
C13—C20—C25—C24	−177.3 (3)	C43—C44—C46—C47	166.2 (3)
C14—C8—C9—N3	179.9 (3)	C44—N39—C40—C24	−177.8 (3)
C14—C8—C9—C10	−3.3 (4)	C44—N39—C40—C41	1.8 (5)
C14—C15—C16—C17	1.5 (5)	C44—C46—C47—C48	−179.6 (4)
C15—C14—C19—C18	0.3 (4)	N45—C46—C47—C48	0.7 (6)
C15—C16—C17—C18	0.2 (5)	C46—N45—C50—C49	0.3 (5)
C16—C17—C18—C19	−1.5 (5)	C46—N45—C50—C51	−177.3 (3)
C17—C18—C19—C14	1.3 (5)	C46—C47—C48—C49	−1.0 (6)
C19—C14—C15—C16	−1.7 (4)	C47—C48—C49—C50	1.0 (6)
C20—C21—C22—C23	−0.2 (4)	C48—C49—C50—N45	−0.6 (6)
C20—C21—C22—C27	−175.9 (3)	C48—C49—C50—C51	176.9 (4)
C21—C20—C25—C24	0.7 (4)	C50—N45—C46—C44	180.0 (3)
C21—C22—C23—C24	−0.5 (4)	C50—N45—C46—C47	−0.3 (5)
C21—C22—C27—N26	156.6 (3)	C52^ii^—C52—O53—C54	172 (2)
C21—C22—C27—C28	−21.8 (4)		

Symmetry codes: (i) −*x*, −*y*+1, −*z*+1; (ii) −*x*+1, −*y*+2, −*z*+1.
